# Biological imprint of education and rights‐based brain capital

**DOI:** 10.1002/alz.70222

**Published:** 2025-05-02

**Authors:** Agustin Ibanez, Temitope Farombi

**Affiliations:** ^1^ Latin American Brain Health Institute (BrainLat) Universidad Adolfo Ibañez, Diagonal Las Torres 2640, Peñalolén, Santiago, Región Metropolitana, CP 7941169 Santiago de Chile Chile; ^2^ Cognitive Neuroscience Center Universidad de San Andrés, Vito Dumas 284, Victoria, B1644BID Buenos Aires Argentina; ^3^ Global Brain Health Institute (GBHI) Trinity College Dublin, Lloyd Building, College Green Dublin 2, D02 PN40 Dublin Ireland; ^4^ Neurology Unit, Chief Tony Anenih Geriatric Center University College Hospital, University College Hospital, Agodi Ibadan, Oyo State, 200212 Ibadan Nigeria

**Keywords:** aging, brain capital, dementia, education, human rights

## 1 INTRODUCTION

Recent findings on the biological embedding of educational disparities in aging and dementia, evidence that access to high‐quality education is both a human rights imperative and a critical public health strategy. Education quantity and quality and related factors shape cognitive health and vulnerability to dementia. These factors are particularly salient in low‐ and middle‐income countries, where austerity policies and systemic disparities frequently compromise brain capital. We advocate for a transdisciplinary approach linking education, social justice, and neuroscience within a rights‐based framework. Addressing structural determinants through education policy can promote healthy brain aging and reduce inequities.

### 1.1 Biological embeddings and rights‐based consequences of education

We commend Timothy Daly ^1^ for highlighting recent crucial findings ^2^ regarding the biological embedding of educational disparities. Daly commentary underscores a powerful and timely message: equitable access to education across the Americas—and globally—is both a matter of human rights and a determinant of national brain health and societal resilience.

We now have robust neuroimaging evidence linking insufficient education with reduced brain volume and disrupted neural connectivity in later life and in dementia ^2^. These findings provide a compelling neurobiological basis for understanding how early‐life educational disparities leave lasting imprints on brain health trajectories. However, the issue is more complex than simply the number of years spent in school. Educational attainment alone does not capture the full picture ^3^. The quality of education ^4^, the social determinants of health ^3^, and other broader structural inequities ^5^ critically shape the biological pathways through which literacy—or lack thereof—affects aging and cognitive decline. The quality and contextual relevance of education, especially in underserved settings ^6^, significantly influence cognitive stimulation, neural development, and resilience against unhealthy aging.

In many regions, especially in low‐ and middle‐income countries and across parts of Latin America and Africa, the right to education is further undermined by austerity policies, fragile public systems, and entrenched systemic inequities. These structural forces erode access to quality education, compromising the dignity of individuals by depriving them of opportunities for social mobility and health equity ^6^, ^7^. When children grow up in environments where basic educational infrastructure is lacking, where teachers are underpaid, or where gender, ethnic, or socioeconomic discrimination limits access, the downstream consequences for brain health and aging are profound.

Education interacts dynamically with other determinants of health. Studies have shown that lifestyle factors, demographic variables, and societal instability are all powerful predictors of cognitive functioning and brain health, especially in developing countries ^3^, ^8^. The relationship between household income and education is notably bidirectional ^9^. Higher‐income households are more likely to access and afford high‐quality educational opportunities, while access to quality education enhances individuals’ lifetime earning potential. This mutual reinforcement forms a feedback loop that deepens inequality across generations ^6^. Individuals in low‐income households often face multiple concurrent barriers that compound the effects of educational disparities. These include limited access to proper nutrition, essential healthcare services, and safe living conditions—all of which are known to affect cognitive development and brain health across the lifespan ^5^. Without addressing these interconnected factors, interventions focused solely on improving educational metrics risk falling short of meaningful impact.

From this broader perspective, protecting and enhancing the quantity and quality of education must be seen simultaneously as a fundamental human right and a powerful public health strategy. Health promotion cannot be disentangled from the social conditions in which people are born, grow, learn, and age ^10^. Education, therefore, should be understood as both a driver and a product of health equity. A comprehensive rights‐based approach must integrate these dimensions to fully capture how education contributes not just to individual dignity, but also to collective well‐being and cognitive health across populations.

The fields of neuroscience and brain health are well‐positioned to contribute to this agenda by advocating for a transdisciplinary framework that bridges the domains of education, human rights, and health. Scientific evidence now supports what many communities have long intuited: that educational disadvantage is a form of health injustice. A rights‐based framework that recognizes the multifactorial pathways linking disparities, education, and brain health ^8^ can offer a transformative narrative—one that reframes cognitive decline and dementia not as inevitable biological outcomes, but as partially preventable manifestations of social inequality.

We call on researchers, clinicians, and policy‐makers to embrace a broader, systems‐level perspective that links structural determinants, education, and brain health. Future prevention strategies must explicitly address the transdisciplinary connections between neuroscience, social policy, and human rights. In doing so, they must not overlook the structural barriers that hinder access to education and health, especially in the regions most affected by inequality and austerity.

## CONFLICT OF INTEREST STATEMENT

TF is supported by an Atlantic Fellowship at the Global Brain Health Institute (GBHI) at Trinity College Dublin. AI is supported by grants from CONICET; ANID/FONDECYT Regular (1210195 and 1210176 and 1220995); ANID/FONDAP/15150012; ANID/PIA/ANILLOS ACT210096; FONDEF ID20I10152, ANID/FONDAP 15150012; Takeda CW2680521 and the MULTI‐PARTNER CONSORTIUM TO EXPAND DEMENTIA RESEARCH IN LATIN AMERICA [ReDLat, supported by Fogarty International Center (FIC), National Institutes of Health, National Institutes of Ageing (AG075775, AG057234, AG082056 and AG083799, CARDS‐NIH 75N95022C00031), Alzheimer's Association (SG‐20‐725707), Rainwater Charitable Foundation – The Bluefield project to cure FTD, and Global Brain Health Institute)]. The views expressed are those of the authors and not necessarily those of the stakeholders. Author disclosures are available in the Supporting Information.

## Supporting information



Supporting Information
